# New Strategy for the Persistent Photocatalytic Reduction of U(VI): Utilization and Storage of Solar Energy in K^+^ and Cyano Co‐Decorated Poly(Heptazine Imide)

**DOI:** 10.1002/advs.202205542

**Published:** 2022-12-13

**Authors:** Jingjing Wang, Ping Li, Yun Wang, Ziyi Liu, Dongqi Wang, Jianjun Liang, Qiaohui Fan

**Affiliations:** ^1^ Northwest Institute of Eco‐Environment Resources Chinese Academy of Sciences Lanzhou 730000 P. R. China; ^2^ Key Laboratory of Petroleum Resources Gansu Province Lanhzou 730000 China; ^3^ State Key Laboratory of Fine Chemicals Liaoning Key Laboratory for Catalytic Conversion of Carbon Resources School of Chemical Engineering Dalian University of Technology Dalian 116024 P. R. China; ^4^ CAS Key Laboratory Nuclear Radiation & Nuclear Energy Technology and Multidisciplinary Initiative Center Institute of High Energy Physics Chinese Academy of Sciences Beijing 100049 P. R. China

**Keywords:** around‐the‐clock catalysis, charge storage, extraction, reduction, uranium

## Abstract

The photocatalytic conversion of soluble U(VI) into insoluble U(IV) is a robust strategy to harvest aqueous uranium, but remains challenging owing to the intermittent availability of solar influx and reoxidation of U(IV) without illumination. Herein, a dual platform based on K^+^ and cyano group co‐decorated poly(heptazine imide) (K‐CN‐PHI) is reported that can drive persistent U(VI) extraction upon/beyond light. K‐CN‐PHI achieves the photocatalytic reduction of U(VI) with a reaction rate of 0.89 min^−1^, being 47 times greater than that over pristine carbon nitride (PCN). This system can further be triggered by light to form long‐living radicals, driving the reduction of U(VI) in the dark for over 3 d. The flexible structural K^+^ as counterions stabilize the electrons trapped by cyanamide groups, enabling the long lifetime of the generated radicals. The results collectively prove K‐CN‐PHI to be a novel and efficient photocatalyst enabling persistent U(VI) extraction around the clock, and broadening the practical applications of the photocatalytic extraction of U(VI).

## Graphical abstract

K^+^ and cyano group co‐decorated poly(heptazine imide) (K‐CN‐PHI) is developed for uranium extraction around‐the‐clock. Under illumination, the K‐CN‐PHI system shows excellent photocatalytic ability for the reduction of U(VI). In the meantime, it can harvest and convert excess solar energy into long‐living polymeric radicals, which drives the reduction of U(VI) in the dark.

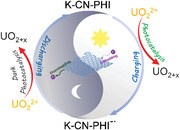

## 1 Introduction

The sustainable development of nuclear power is a highly promising strategy for “zero‐emission credits”.^[^ ^1^ ^]^ As the strategic resource for the nuclear industry, uranium is crucial for promoting the feasibility of sustainable nuclear energy.^[^ ^1^, ^2^ ^]^ Compared with terrestrial uranium reserves, the ocean contains significantly more uranium (≈4.5 billion tons), which can theoretically satisfy the demand for uranium supplies for centuries. Therefore, the recovery of uranium from seawater is considered one of the seven chemical separations with potential to change the world.^[^ ^3^, ^4^ ^]^ Meanwhile, the uranium‐containing wastes generated during nuclear industry‐related activities present potential threats on account of their radioactive and chemically toxic nature.^[^ ^5^, ^6^ ^]^ Thus, from the perspectives of strategic resource recovery and ecological environmental safety, newer and more effective technologies should be developed to separate and recover uranium from aqueous solution.

Heterogeneous photocatalysis, during which highly soluble U(VI) is reduced to immobile U(IV), has recently emerged as a thriving strategy for U(VI) separation from waters.^[^ ^7^ ^]^ However, the reported catalysts can only function under light irradiation.^[^ ^6^, ^7^, ^8^, ^9^, ^10^, ^11^, ^12^, ^13^, ^14^, ^15^, ^16^, ^17^, ^18^, ^19^, ^20^, ^21^, ^22^, ^23^, ^24^, ^25^, ^26^, ^27^, ^28^, ^29^ ^]^ Once light input is withheld, the reaction process immediately ceases owing to the inability of the catalysts to generate charge carriers, which impedes the continuous extraction of U(VI) at night. Moreover, when light irradiation is ceased, the photocatalytically reduced products could be re‐oxidized, resulting in the redissolution of extracted uranium.^[^ ^17^ ^]^ This intermittent reaction process greatly limits the applications of photocatalysis in uranium extraction. Given these considerations, developing methods that could achieve persistent photocatalysis with satisfactory reduction ability in both the presence and absence of light is of significant importance. Traditional long‐afterglow phosphor materials, a type of energy‐storage materials, have been applied to activate photocatalysts by emitting long‐lasting phosphorescence in the dark even after the cessation of light excitation.^[^ ^30^ ^]^ However, most long‐afterglow phosphors used in photocatalytic systems are inherently challenged by ultrafast triplet exciton deactivation, low fluorescence intensity, and the high cost of rare‐earth elements.^[^ ^31^ ^]^ Good catalytic activity in the dark was recently achieved by endowing the photocatalysts with post‐illumination “memory” activity via compositing with materials such as TiO_2_/WO_3_,^[^ ^32^ ^]^ TiO_2_/Cu_2_O,^[^ ^33^ ^]^ *n*‐TiO_2_/PdO,^[^ ^34^ ^]^ and TiO_2_/SnO_2_.^[^ ^35^ ^]^ The photogenerated electrons in these composites are trapped through the valence transformation of metal ions in the photocatalysts (e.g., Ti^4+^–Ti^3+^, Pd^2+^–Pd^0^, W^6+^–W^(6‐x)^, and Sn^4+^–Sn^2+^). However, the lifetimes are fairly short, ranging from only several minutes to a few hours. Compared with these photocatalysts, poly(heptazine imide) (PHI), a carbon nitride derivative, is able to form a long‐life photo‐reduced state for days after light irradiation.^[^ ^36^, ^37^, ^38^, ^39^, ^40^, ^41^ ^]^ However, the potential mechanistic understanding of dark photocatalysis remains incompletely explored.

In this study, K^+^‐doped and cyanamide‐functionalized PHI (K‐CN‐PHI) was fabricated and applied to the photocatalytic reduction of U(VI), and the relevant reaction mechanism was investigated in detail. Owing to the unique structure and intriguing photophysical properties of the photocatalyst, continuous U(VI) extraction was achieved in both light and dark conditions. The results of our work can provide new guidelines for the development of energy‐storage photocatalysts capable of efficient round‐the‐clock U(VI) extraction.

## 2 Results and Discussion

### 2.1 Synthesis and Characterization

**Figure 1:**
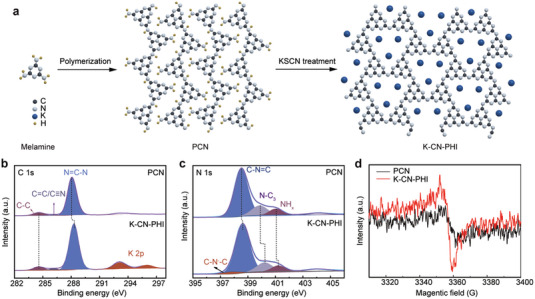
Characterization and structures of the photocatalysts. a) ideal structures, b) C 1s and c) N 1s XPS spectra, and d) EPR of PCN and K‐C‐PHI.

K‐CN‐PHI was synthesized from the post‐polymerization of PCN in KSCN salt, this treatment leads to an obvious structural rearrangement of the product (**Figure** **1**a). Different from the typical X‐ray diffraction (XRD) peaks of pristine carbon nitride (PCN) (Figure S1a, Supporting Information),^[^ ^42^ ^]^ K‐CN‐PHI revealed the obvious shift of the (002) diffraction peak to 27.9°, indicating a decreased interlayer distance from 3.25 to 3.19 Å. In addition, the diffraction peak of the (100) facet disappeared, and two new peaks at ≈8.0° and 9.9°, which could be assigned to the (−110) and (010) facets of PHI, respectively, appeared.^[^ ^43^ ^]^ This observation indicates the full transformation of heptazine units to form the PHI network after KSCN treatment. Moreover, K^+^ and cyano groups were successfully incorporated into the PHI framework, as verified by the symmetric and asymmetric vibrations of NC_2_ bands of C_2_N^−^K^+^ group at 911 and 1,010 cm^−1^, as well as the typical —C≡N band at 2,150 cm^−1^ in the Fourier transform infrared (FT‐IR) spectrum of K‐CN‐PHI (Figure S1b, Supporting information).^[^ ^44^ ^]^ This strong electron‐withdrawing group could act as an efficient electrons‐trapping center to promote electron delocalization and suppress carrier recombination. The chemical environment afforded by K^+^ ions in the PHI framework was elucidated by density functional theory (DFT) calculations. In the most stable configuration of the K‐CN‐PHI structure (Figure S2, Supporting Information), K^+^ ions were stabilized at the center of four pyridinic nitrogen atoms from two heptazine units. The solid‐state ^13^C nuclear magnetic resonance (NMR) and X‐ray photoelectron spectroscopy (XPS) analysis provide further insights into the structure information. The two main resonances at ≈157 and ≈163 ppm in the ^13^C NMR spectrum of K‐CN‐PHI (Figure S3, Supporting Information) can be attributed to the carbon atoms in C‐N_3_ and C‐N_2_(NH_x_) of the PHI skeleton.^[^ ^45^, ^46^ ^]^ In the high resolution C 1s spectra (Figure 1b), the two new peaks located at 292.8 and 295.6 eV could be attributed to K 2p_1/2_ and 2p_3/2_, respectively.^[^ ^47^ ^]^ The intensified C=N/C≡N peak at 286.4 eV (Figure 1b) could be interpreted as additional evidence of the formation of the —C≡N group in K‐CN‐PHI.^[^ ^48^ ^]^ The N 1s peak at 397.8 eV (Figure 1c) is ascribed to deprotonated N^—^ atoms, which could be balanced by K^+^ ions via nitrogen–metal donor–acceptor interactions.^[^ ^49^ ^]^ The modification of K^+^ and strong electron‐withdrawing cyano groups alerted the surface electronic distribution in K‐CN‐PHI, causing the binding energies (BEs) of the C 1s and N 1s peaks to shift slightly toward higher values.^[^ ^50^, ^51^ ^]^ In addition, the strong electron‐withdrawing cyano groups in K‐CN‐PHI also caused an enhanced electron paramagnetic resonance (EPR) signal (Figure 1d), revealing a much higher number of unpaired electrons within the *π*‐conjugated structure.^[^ ^52^ ^]^ The rearranged electronic structure make K‐CN‐PHI exhibits an obviously different micromorphology feature, as confirmed by scanning electron microscope (SEM) and transmission electron microscope (TEM) (Figure S4, Supporting Information). Compared with PCN, which featured an irregular and multilayered structure (Figure S4a,c, Supporting Information), K‐CN‐PHI showed multiple separated sheets with a smaller particle size (Figure S4b,d, Supporting Information) and thereafter a higher Brunauer−Emmett−Teller (BET) surface area (93.50 m^2^ g^−1^, 5.9 times higher than PCN) (Figure S1c,d, Supporting information). These characteristics enable K‐CN‐PHI more abundant active sites and facilitate mass transport toward efficient uranium photocatalysis.^[^ ^53^ ^]^

**Figure 2:**
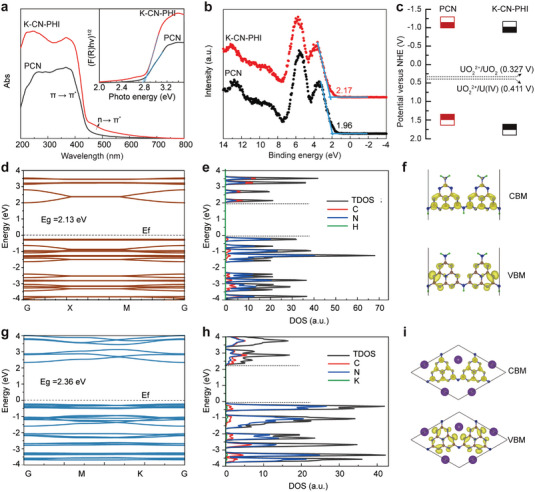
Band structures of the photocatalysts. a) UV–vis DRS spectra and (inset) Tauc plots, b) Valence band (VB)‐XPS spectra, and c) estimated band structures of PCN and K‐CN‐PHI. d,e) Calculated (d) total and (e) partial density of states of PCN. f,g) Calculated (f) total and (g) partial density of states of K‐CN‐PHI. h,i) Calculated CBM and VBM distributions of (h) PCN and (i) K‐CN‐PHI.

The UV–vis diffuse reflectance spectra (DRS) spectrum of K‐CN‐PHI indicated enhanced light‐harvesting ability in the UV and visible regions compared with PCN (**Figure** **2**a). For K‐CN‐PHI, in addition to the electron transition of *π*→π* below 450 nm in the conjugated aromatic system, an additional absorption band in the region of 450–600 nm, which could be ascribed to the transition of n→π* induced by lone‐pair electrons at N‐defect sites (C≡N), was observed.^[^ ^52^, ^54^ ^]^ The band structure of K‐CN‐PHI was also adjusted, with a slightly increased bandgap (*E*_g_) that is caused by the quantum confinement effect owing to the decreased interlayer distance (insert in Figure 2a).^[^ ^55^ ^]^ Valence band (VB)‐XPS analysis (Figure 2b) revealed VB potentials (*E*_VB_) of 1.51 and 1.73 V (vs a normal hydrogen electrode [NHE], based on the formula in Supporting Information) for PCN and K‐CN‐PHI, respectively. As a result, the conduction band (CB) potentials (*E*_CB_) of PCN and K‐CN‐PHI were determined to be −1.14 and −0.92 V (vs NHE), respectively, providing a thermodynamic driving force for the reduction of U(VI) (Figure 2c).

The density‐functional theory (DFT) calculations were employed to clarify the electronic band structures of PCN and K‐CN‐PHI further. As shown in Figure 2d,g, the calculated *E*_g_ value was smaller than the experimental value, which could be attributed to the underestimation and limitations of the computer calculations.^[^ ^56^ ^]^ In addition, the bandgap of K‐CN‐PHI (2.36 eV, Figure 2g) was calculated to be larger than that of PCN (2.13 eV, Figure 2d), which agrees well with the DRS results. The total and partial density of states of PCN and K‐CN‐PHI were also calculated to demonstrate the electron cloud distribution and contribution of each element. For both cases, N orbitals dominated the VB maximum (VBM), and the CB minimum (CBM) contained the contributions of the C and N orbitals (Figure 2e–i). In particular, for K‐CN‐PHI, the contribution of K orbitals led to an obvious upward shift of the VBM (Figure 2h), which supports the VB‐XPS spectra. The isosurface plots of different charge densities of K‐CN‐PHI implied an obvious charge redistribution, in which K atom loss 0.89 e^−^ to adjacent atoms, and N1 and N2 atoms gain 0.8 and 0.84 e^−^, respectively (Figure S5, Supporting Information). This finding implies that K^+^ was stabilized in the PHI framework via the strong electrostatic attraction between the electron‐rich N atoms and K^+^, which matches with the XPS and FT‐IR analysis well. The presence of K^+^ altered the local charge distribution in K‐CN‐PHI, and its electron mobility and charge transfer kinetics were expected to be improved.

**Figure 3:**
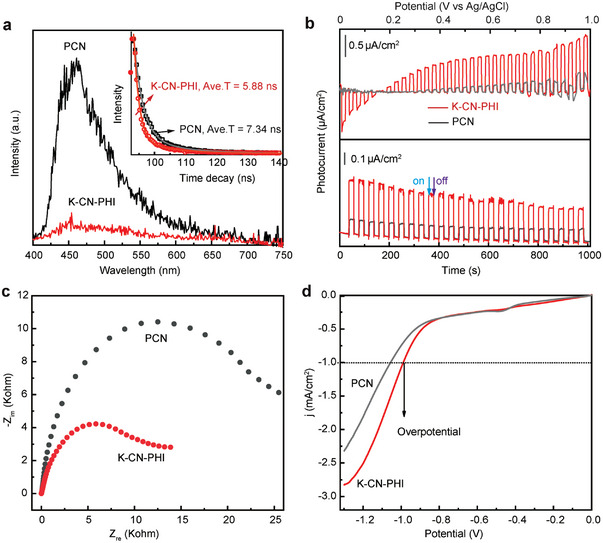
Photoelectric properties of the photocatalysts. a) Fluorescence spectra (inset: time‐resolved fluorescence decay spectra), b) potential bias‐dependent photocurrent measurements under intermittent irradiation (after baseline deduction) and photocurrent transient responses at 0.5 V (vs Ag/AgCl), c) EIS spectra, and d) LSV curves of PCN and K‐C‐PHI.

As shown in **Figure** **3**a, K‐CN‐PHI showed strongly quenched photoluminescence (PL) emission signals, indicating a lower recombination probability than PCN. The time‐resolved PL decay spectra of K‐CN‐PHI (insert in Figure 3a) showed faster PL decay kinetics, as well as a reduction in average emission lifetime from 7.34 to 5.88 ns. This reduction in emission lifetime indicates the occurrence of accelerated exciton dissociation and transfer in K‐CN‐PHI.^[^ ^57^, ^58^ ^]^ The photocurrent–voltage curves and transient photocurrent measurements exhibited significant improvements in the photocurrent responses and photon‐to‐electron conversion efficiency of K‐CN‐PHI (Figure 3b), leading to the highly effective separation of photogenerated electrons and holes. The electrochemical impedance spectra (EIS) of K‐CN‐PHI suggested progressive decreases in charge transfer resistance (Figure 3c), thereby reflecting its superior charge separation and transfer efficiency. The linear sweep voltammetry (LSV) curves shown in Figure 3d indicate that, in comparison with PCN, K‐CN‐PHI exhibits a higher cathodic current density and lower overpotential (*η*_i_), resulting in higher activity for the photocatalytic reduction reaction.

### 2.2 Photocatalytic Activity Toward U(VI)

**Figure 4:**
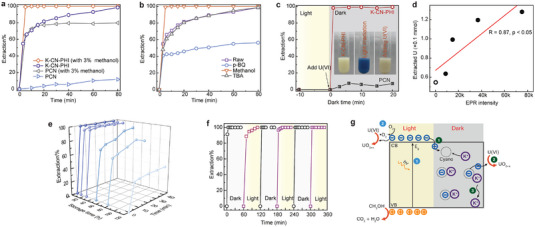
Light and dark driven reduction of U(VI). a) Photocatalytic kinetics of U(VI) on PCN and K‐CN‐PHI. b) Effect of different scavengers on the extraction efficiency of K‐C‐PHI toward U(VI). c) Dark reaction kinetics of U(VI) over PCN and K‐CN‐PHI obtained by spiking U(VI) into pre‐irradiated suspensions (pre‐irradiation time = 10 min). d) Extraction efficiency of U(VI) on K‐CN‐PHI versus EPR intensity. e) Dark reaction kinetics of U(VI) on K‐CN‐PHI in different post‐irradiation periods. f) Alternating reactions of U(VI) on K‐CN‐PHI over three dark/light cycles. In the dark process, 0.1 mm U(VI) was added to the blue K‐CN‐PHI suspension, and the mixture was reacted for 60 min. Then, 0.1 mm U(VI) was added to the suspension, and the mixture was subjected to light irradiation (light process). After the complete removal of U(VI), the suspension turned blue once more, and the light source was removed. The suspension was subjected to two more cycles of the dark/light process. g) Schematic diagram of the mechanisms for the photo‐ and dark reduction of U(VI) on K‐CN‐PHI.

In the dark, K‐CN‐PHI adsorbed 72% of the U(VI) in the test solution, whereas PCN harvested <3% of the available U(VI) (Figure S6a, Supporting Information). The markedly higher adsorption ability of the former may be ascribed to its smaller particle size, larger surface area, and additional functional groups, all of which provide more reactive sites for U(VI). Photocatalytic uranium extraction was performed as depicted in **Figure** **4**a. K‐CN‐PHI showed excellent photocatalytic activity toward U(VI) reduction, where 100% of the U(VI) was extracted within ≈5 min. The reaction rate (*k*) of U(VI) over K‐CN‐PHI was determined to be 0.89 min^−1^, being 46.8 times higher than that over PCN (0.019 min^−1^) (Figure S6b, Supporting Information)_._ To evaluate the performance for uranium removal in environment, the photocatalytic U(VI) reduction was tested by varying the concentration of U(VI) from 3.5 ppb to 10 ppm, and almost all of the U(VI) could be eliminated within 10 min (Figure S6c, Supporting Information). The excellent photocatalytic performance of K‐CN‐PHI toward U(VI) extraction was also verified when the concentration of U(VI) was up to 1.0 mm (Figure S6d, Supporting Information); specifically, all of the U(VI) in solution could be eliminated at each run. Further increasing the U(VI) amount in the system (spiking 0.2 mm U(VI) for 7 times), the photocatalytic U(VI) reduction gradually decreased, and still 62% of uranium was removed at the last run (Figure S6e, Supporting Information), demonstrating the high photocatalytic ability of K‐CN‐PHI toward U(VI). Even without methanol as a hole scavenger, U(VI) could still be completely extracted within 80 min (Figure 4a). By contrast, PCN presented a sluggish and low uranium harvest (Figure 4a).

The photoinduced active radicals involved in the photocatalytic system were further determined by EPR. From Figure S7a (Supporting Information), no signals were detected without light irradiation. After illumination, the typical signals of DMPO‐•O_2_^−^ and DMPO‐•OH adducts could be observed, indicating the formation of •O_2_^−^ and •OH radicals. The much stronger signal intensity of K‐CN‐PHI than that of PCN demonstrates the powerful light‐driven radical species evolution ability. *Tert*‐butyl alcohol (TBA), *p*‐benzoquinone (*p*‐BQ), and methanol were employed to scavenge photoproduced •OH, •O_2_^−^, and *h* ^+^, respectively. The photocatalytic efficiency of K‐C‐PHI was considerably inhibited by *p*‐BQ but not influenced by TBA, thus revealing that •O_2_^−^ dominantly controlled the photocatalytic U(VI) reduction, whereas •OH did not influence the photocatalytic reaction (Figure 4b). Separately, the photocatalytic kinetic was enhanced by the addition of methanol because its oxidization forms •CO_2_^−^ and the trapping of *h* ^+^ promotes the use of electrons by molecular oxygen to form •O_2_^−^.^[^ ^26^ ^]^

In view of the above results, the superior photoactivity of the K‐CN‐PHI nanosheets developed in this work could be associated with enhanced adsorption affinity, improved optical absorption, optimized electronic band structures for charge migration and separation, and greater ability to produce reductive radicals.

### 2.3 Dark Photocatalysis of U(VI) over K‐CN‐PHI

**Figure 5:**
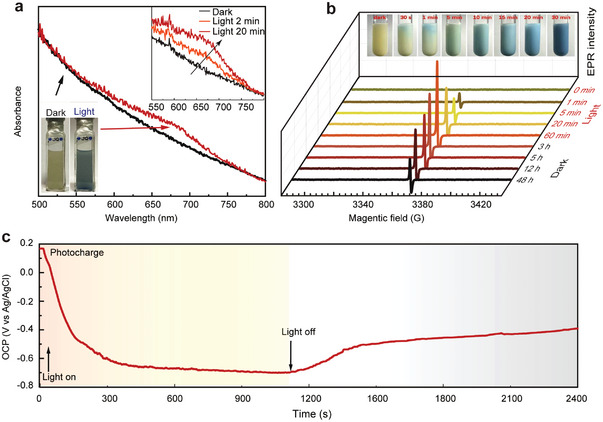
Mechanistic insights into the formed long‐living radicals. a) UV–vis absorption spectra of the K‐CN‐PHI suspension before and after light irradiation (insert: UV–vis absorption spectra under different irradiation time). b) EPR signals of the K‐CN‐PHI suspension collected under different irradiation times and dark times. c) Open circuit potential (OCP) measurements of K‐CN‐PHI collected under light irradiation and in the dark after irradiation.

It is interesting to note that, in the depletion or complete absence of U(VI), the consistent illumination of the K‐CN‐PHI suspension induced an instantaneous color change from light yellow to blue (inset in **Figure** **5**a). This color change was also reflected in the UV–vis absorption spectra, which shows the appearance of a broad absorption peak at 550–750 nm (Figure 5a). EPR spectroscopy was employed to obtain deeper insights into the nature of this blue suspension. As shown in Figure 5b, the light‐generated blue K‐CN‐PHI suspension exhibited a distinct singlet paramagnetic EPR signal with g‐factor of 2.0049, suggesting the formation of an organic radical.^[^ ^59^ ^]^ Moreover, this type of radical appeared immediately within 1 min of light illumination. Longer irradiation times led to stronger EPR signals, corresponding to the darkening of the color of suspension (Figure S8, Supporting Information). It should be mentioned that when the generated free radicals got saturated, the intensity of the EPR signals slightly decreased by further prolonging the irradiation time (Figure S9, Supporting Information), which may be due to the consumption of the free radicals by some oxidative species (e.g., •OH) formed during the irradiation process. Surprisingly, this blue suspension showed remarkable long‐term stability over several days in the dark (Figure S10, Supporting Information), and a high EPR signal was obtained even after 48 h (Figure 5b). These findings reveal the long lifetime of the organic radicals generated by K‐CN‐PHI.

The radicals formed are also reflected by the potential changes on the K‐CN‐PHI electrode. As shown in Figure 5c, under illumination, the radicals accumulated on the K‐CN‐PHI electrode decreased the potential, which plateaued at −700 mV (vs Ag/AgCl). Moreover, the color of the electrode gradually changed from pristine yellow to bright blue (Figure S11a, Supporting Information). When the light source was switched off, the electrode potential slightly increased to −500 mV (vs Ag/AgCl) and remained at this value for a relatively long time (over 1,000 s after cessation of illumination) (Figure 5c). This observation reflects the stabilization of the stored radicals in the dark period following illumination. Moreover, the highly negative potential indicates that the colored radical anions on K‐CN‐PHI are highly reductive and, thus, may be expected to drive the reduction of U(VI) in the dark.

Based on above analyses, the dark reaction of U(VI) was evaluated by spiking 0.1 mm U(VI) into a pre‐irradiated blue K‐CN‐PHI suspension. As shown in Figure 4c, ≈100% U(VI) was eliminated from solution within 1 min by the charged K‐CN‐PHI. In addition, the blue coloration of the solution rapidly disappeared, and the catalyst surface turned gray owing to the deposition of reduced uranium products (Figure 4c; Figure S11b, Supporting Information). In sharp contrast, only 7% of the U(VI) in solution was extracted by pre‐irradiated PCN, totally via adsorption (Figure 4c). Compared to the data reported in literature (Table S1, Supporting Information), K‐CN‐PHI showed superior performance in both light and dark environment. When further increasing uranium concentration to 0.2 mm, 82% of U(VI) could be removed within 1 min, indicating the high dark reduction ability of the formed radicals for each run (78.1 mg g^−1^, Figure S6f, Supporting Information). Even though the number of the radicals is limited for a certain amount of catalyst, a high reduction capacity for uranium can be achieved by multiple irradiations. As exhibited in Figure S6g (Supporting Information), almost all of the U(VI) could be removed after four runs, and the capacity for uranium reduction was calculated to be >190.4 mg g^−1^. Notably, as reflected by the intensity of the detected EPR signals, the reaction efficiency depended on the number of generated radicals. Figure 4d shows a reasonable positive correlation between the extraction capacity of the photocatalyst for U(VI) and its EPR signal intensity (*R* = 0.87, *P* < 0.05). When the dark reaction of U(VI) was completed, the EPR signal (Figure S12a, Supporting Information) and UV–vis absorption peak at 450–700 nm (Figure S12b, Supporting Information) completely disappeared, confirming that the accumulated radicals were consumed by U(VI) under dark conditions. These observations suggest that the nascent radicals were mainly responsible for U(VI) reduction in the dark. Figure 4e shows that the reduction of U(VI) over K‐CN‐PHI was maintained even after 72 h; moreover, the blue coloration of the solution nearly completely disappeared 120 h after light irradiation, at which point 43% of the available U(VI) was removed mainly by adsorption. These results confirm that the longevity of radicals could maintain the photocatalytic reduction ability of the catalyst in the dark.

As shown in Figure 4f, K‐CN‐PHI showed high efficiency for U(VI) extraction over at least three dark–light cycles. Under light irradiation, the photoinduced electrons are stored in the form of stable radicals when all of the available U(VI) is photocatalytically reduced. Under dark conditions, the formed radicals undergo rapid discharging to continue U(VI) reduction. Thus, the K‐CN‐PHI nanosheets possess persistent photocatalytic activity in both lightness and darkness, meanwhile providing an excellent platform that could avoid the reoxidation of reduced uranium products.

### 2.4 Conversion Mechanisms of U(VI)

**Figure 6:**
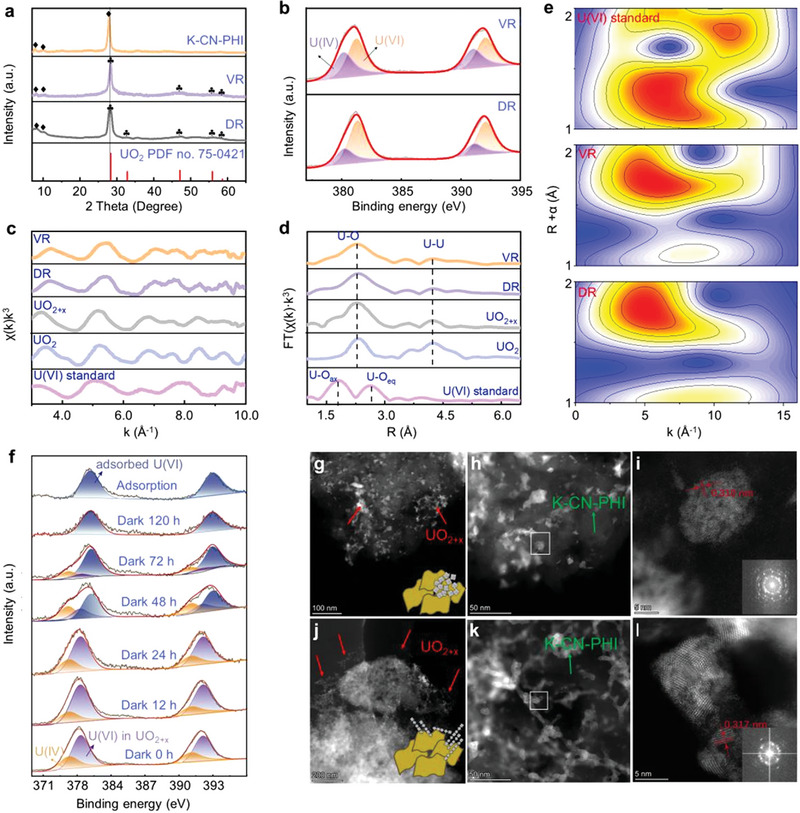
Mechanism studies for the conversion of U(VI). a) XRD patterns, b) U 4f XPS spectra collected after the light and dark reactions. c) U LIII‐edge *k* ^3^‐weighted EXAFS spectra, and d) corresponding Fourier transforms (R space) of the reference samples (data of UO_2_ and UO_2+x_ product from Fletcher et al.^[^ ^70^ ^]^ and Bone et al.,^[^ ^63^ ^]^ respectively) and reaction products after light and dark reduction. e) Wavelet transforms of the EXAFS spectra of U(VI) standard (top), and the reaction products after light (middle) and dark reduction (bottom). f) U 4f XPS spectra collected after different dark delay times. g–l) Scanning transmission electron micrographs (STEM) of the reaction products after (g–i) light and (j–l) dark reduction.

XRD, XPS, and X‐ray absorption spectroscopy (XAS) were employed to decipher the mechanisms of the visible‐light photocatalytic reduction (VR) and dark reduction (DR) of U(VI) over K‐CN‐PHI. The XRD patterns of the VR and DR samples in **Figure** **6**a showed additional typical peaks of uraninite structures (UO_2_ or UO_2+x_, 0 < x < 0.25), both samples also changed in color to dark gray after the reaction (Figure S11b, Supporting Information).^[^ ^17^, ^26^, ^27^, ^60^ ^]^ The XPS spectra of the VR and DR samples confirmed the formation of a U(VI)/U(IV) mixture (Figure 6b). Comparison of the BEs of the U(VI) species revealed that the BEs of U(VI) in the reduction products (381.3 (U 4f_5/2_) and 392.1 eV (U 4f_7/2_)) were lower compared with that of the adsorbed U(VI) (382.2 and 393.0 eV, Figure 6f and Figure S13a, Supporting Information), indicating that U(VI) was reduced to form UO_2+x_ but not the mixture of UO_2_ and adsorbed uranyl.^[^ ^27^, ^60^, ^61^, ^62^ ^]^ The transformation of U(VI) over K‐CN‐PHI by the blue‐colored radicals in the dark was verified to be similar to that under light, with the products consisting of UO_2+x_. In Figure S13b (Supporting Information), the first derivative U L_III_‐edge of the VR and DR samples showed similar absorption edges, and both the energy positions were lower than that of U(VI), which suggests similar reduction products. As indicated by the *k* ^3^‐weighted extended X‐ray absorption fine structure (EXAFS) spectra and corresponding Fourier‐transformed (FT) EXAFS signals (Figure 6c,d), the average local chemical environment of U in the VR and DR samples was quite different from that of U(VI) fingerprints but more similar to that of UO_2+x_.^[^ ^63^, ^64^ ^]^ The wavelet transforms (WT)‐EXAFS contour plots (Figure 6e) of both VR and DR samples exhibited sole intensity maximum at (1.8 Å, 5.0 Å^−1^) (uncorrected for phase shifting) at a given range, which is obviously distinguished from the U(VI) standard, corresponding to the U—O bonds of U(IV).^[^ ^63^, ^64^ ^]^ The transformation of U(VI) over the charged K‐CN‐PHI at different darkness exposure times was determined by XPS. Given the long lifetime of the formed reductive radicals, the obvious reduction of U(VI) occurred for over 72 h in the dark after irradiation (Figure 6f). However, the gradual annihilation of the radicals over time prohibited the complete reduction of U(VI), and the adsorbed U(VI) species appeared on the DR samples when the post‐irradiation time exceeded 48 h (Figure 6f). These findings are in accordance with the results shown in Figure 4e.

High‐angle annular dark‐field scanning transmission electron microscopy (HAADF‐STEM) was applied to identify the structure and localization of reduced UO_2+x_ species. For the VR samples, abundant nanoclusters measuring 5–20 nm in size were found on the K‐CN‐PHI nanosheets (Figure 6g−i; Figure S14a, Supporting Information). The clusters clearly showed lattice fringe spacings close to the (111) facet in UO_2+x_.^[^ ^65^ ^]^ Moreover, the UO_2+x_ clusters appeared to be composed of a large number of disorderly arranged nanoparticles (Figure 6i), and all of the aggregates were anchored to the K‐CN‐PHI surface. By contrast, the DR samples showed a very different product morphology; in this case, the products showed an interlacing network of chains and branches with a diameter of 2–10 nm appearing on or outside of the K‐CN‐PHI surface (Figure 6j,k; Figures S14b and S15, Supporting Information). The high‐resolution images show that the interlaced chains originate from the randomly oriented attachment of single UO_2+x_ nanoparticles (Figure 6l). Similar nanochain structures were reported during the chemical reduction of U(VI) by glutathione (GSH), and the formation of a network‐like UO_2+x_ precipitate was observed after reduction.^[^ ^66^ ^]^ A transient uranium nanowire structure has also been observed during the magnetite‐induced reduction of uranium.^[^ ^67^ ^]^ Therefore, similar reduction products, but completely different reduction pathways, may be involved in the light and dark reductions of U(VI). For the VR samples, U(VI) was reduced by the •O_2_^−^ radicals and electrons generated on the active sites of the K‐CN‐PHI surface (Figure 4g), being similar to the “one radish, one hole” concept. The reduced products agglomerate in nanoclusters on the surface of photocatalyst. For the DR samples, the electron‐abundant cyan groups may act as “electron pumps” to release electrons continuously so that uranium is deposited along the discharge direction and forms a unique chain‐like UO_2+x_ precipitate. In this case, the reduction reaction does not rely on the surface reactive sites; instead, the electron‐abundant cyan groups drive the growth of UO_2+x_ nanoparticles into nanowires and extend out of the K‐CN‐PHI surface.

### 2.5 Mechanism of the Formation of Long‐Living Radicals

It has been proven that the dark photocatalysis of U(VI) could be realized by the formation of long‐living radicals in K‐CN‐PHI. However, the mechanisms of the formation and storage of such blue radicals remain unclear.^[^ ^68^, ^69^ ^]^ Interestingly, the K^+^ ions hosted in the PHI conjugate rings are reversible and could be replaced by NH_4_^+^ via ion exchange by agitating the K‐CN‐PHI powder in NH_4_Cl solution (0.01 m). XPS analysis showed that most of the K^+^ ions were exchanged by NH_4_^+^ to form NH_4_‐CN‐PHI (Figure S16a, Supporting Information). In contrast to K‐CN‐PHI, NH_4_‐CN‐PHI was very weakly blue (Figure S17a, Supporting Information), and its electron paramagnetic resonance (EPR) signals were significantly quenched (Figure S16b, Supporting Information). However, when the NH_4_‐CN‐PHI powder was redispersed into a KCl salt solution, the suspension gradually regained its blue color under light irradiation, and its EPR intensity gradually increased (Figures S16c and S17a, Supporting Information). A similar phenomenon was observed when K^+^ was exchanged with Na^+^ to form Na‐CN‐PHI (Figure S17b, Supporting Information). This result indicates the key role of K^+^ in the formation of blue‐colored radicals. The role of the cyano group is also verified based on DFT calculation (Figure S18a,b and Table S2, Supporting Information). Owing to the good electron withdrawing property of the cyano group, excess electron will accumulate in the nitrogen of cyano group, making the nitrogen in cyano a better nucleophilic and radical reaction site from Fukui functions (f_+_, f_0_). In previously reported solar‐intercalation batteries, such as WO_3_ and MoO_3_, the storage of photoexcited charges was associated with reversibly intercalated cations (e.g., K^+^, Na^+^, or Li^+^). Similarly, in the present system, the photogenerated electrons appeared to be trapped by the electron‐withdrawing cyano groups in the K‐CN‐PHI framework and were balanced by the structural K^+^ ions (Figure 4g). When NH_4_^+^ and Na^+^ are compared, the size of K^+^ appears to be easier to take off and embed into the CN‐PHI framework for charge compensation. Thus, the electrons were well stabilized in the K‐CN‐PHI structure in the form of long‐living organic radicals. When U(VI) is spiked to the system, U(VI) was reduced by the electrons accumulated on cyano groups through direct electron transfer, which is different from the photocatalysis under light (Figure 4g). In the meantime, K^+^ tends to take off from the framework owing to the unbalanced structure caused by the consumption of trapped electrons. This was further proved by the decrease in intensities of the K 2p XPS spectrum and C_2_N^−^K^+^ band after multiple reduction of U(VI) (Figure S19a,b, Supporting Information). Fortunately, although K^+^ was released, it could re‐intercalate into PHI framework upon light irradiation to balance the negative charge, and this led to an excellent cycling performance (Figure 4f; Figure S6g, Supporting Information). Moreover, as proved in Figure S16 (Supporting information), the ability for the dark photocatalysis can even be promoted by simply increasing the concentration of extra K^+^.

## 3 Conclusion

In summary, K‐CN‐PHI was prepared by doping K^+^ into cyanamide‐functionalized PHI for U(VI) enrichment from waters. K^+^ played a critical role in stabilizing the negative‐charged electrons on the cyanamide groups under illumination. This photocatalytic system can overcome the limitations presented by the intermittent availability of sunlight, forming long‐living and highly reductive radicals. The high photocatalytic activity and long‐lasting reactivity of the radicals generated by K‐CN‐PHI enabled the efficient and continuous reduction of U(VI) into insoluble U(IV) around the clock. The reduced U(IV) products are then in situ fixed onto the K‐CN‐PHI surface, which prevents their reoxidation in the dark. These findings can provide a new direction for the development of advanced energy storage polymers for sustainable and efficient uranium resource recycling. This work also confirms, for the first time, the dark photocatalysis toward uranium is indeed possible. Further investigations to improve the storage capacity and stability of the generated polymeric radicals could help extend the applications of the existing materials in dark photocatalysis, for example, for pollutant degradation, heavy‐metal reduction, and bactericidal disinfection, among others. On the other hand, the intrinsic variation of the polymeric framework remains unclear once the accumulated radicals are consumed, which should be critical for the rational design of materials and deserves further exploration.

## 4 Experimental Section

### Synthesis of the Photocatalysts

Polymeric g‐C_3_N_4_ (PCN) was synthesized by heating melamine (6 g) at 550 °C for 4 h (heating rate = 10 °C min^−1^). K‐CN‐PHI was prepared as previously described.^[^ ^41^ ^]^ Briefly, PCN and potassium thiocyanate (KSCN; mass ratio = 1:2) were thoroughly ground and baked at 400 °C for 1 h (30 °C min^−1^) and then up to 500 °C for 30 min (30 °C min^−1^). The reaction product was naturally cooled to 25 °C, and the resultant yellow solids were grounded, washed with copious amounts of deionized water, and freeze‐dried overnight.

### Photocatalysis Test

In a light‐driven photocatalytic process, 6 mg of the catalysts was dispersed into a U(VI) solution (15 mL, 0.2 mm, pH 6.0). N_2_ was then bubbled through the suspension, and the system was simultaneously illuminated by a 300 W Xe lamp (UV filtered out). The suspension was periodically sampled at specific reaction times, separated with a 0.22 µm syringe filter, and finally measured by UV–vis spectrophotometer or the inductively coupled plasma–mass spectrometry (ICP‐MS) (see Supporting Information for further details). In a dark photocatalytic process, catalysts (7.5 mg) were suspended in methanol/water (0.5:15, *v*/*v*). The reaction system was then irradiated under N_2_ atmosphere for 40 min. Illumination was ceased, and the blue suspension formed was kept in the dark (by wrapping aluminum foil) for a certain period of time. A uranyl solution was then injected into the reactor (the concentration of U(VI) in the system equals to 0.1 mm). The detailed descriptions for the reduction capacity of K‐CN‐PHI can be found in the Supporting Information. The subsequent steps were identical to those employed for the light‐driven photocatalytic process. In the alternating light/dark reduction experiment, the kinetics of the reaction system with and without light irradiation was recorded by repeating the experiments three times, first for 30 min under visible‐light and then for 30 min in the dark. The extraction efficiency was calculated by using Equation (1).

(1) $$\mathrm{Extraction}\%=\left(1-C_{t}/C_{0}\right)\times 100\%$$

where *C*_0_ and *C_t_* represent the concentration of U(VI) at the beginning and time *t*, respectively. The methods for characterizing samples and theoretical calculations are provided in the Supporting Information.

## Conflict of Interest

The authors declare no conflict of interest.

## Supporting information

Supporting Information

## Data Availability

The data that support the findings of this study are available from the corresponding author upon reasonable request.
