# Supplementary material for: New Strategy for the Persistent Photocatalytic Reduction of U(VI): Utilization and Storage of Solar Energy in K+ and Cyano Co‐Decorated Poly(Heptazine Imide)
Source: Adv Sci (Weinh). 2022 Dec 13;10(5):2205542. doi: 10.1002/advs.202205542 (PMC9929247; doi:10.1002/advs.202205542)
Supplement: Supplementary file 1 — Supporting Information [file ADVS-10-2205542-s001.pdf]

ADVANCED SCIENCE
Open Access

Supporting Information

for *Adv. Sci.*, DOI 10.1002/advs.202205542

New Strategy for the Persistent Photocatalytic Reduction of U(VI): Utilization and Storage of Solar Energy in K^+ and Cyano Co-Decorated Poly(Heptazine Imide)

Jingjing Wang, Ping Li, Yun Wang, Ziyi Liu, Dongqi Wang, Jianjun Liang and Qiaohui Fan**

Supporting Information

New strategy for the persistent photocatalytic reduction of U(VI): Utilization and storage of solar energy in K^+ and cyano co-decorated poly(heptazine imide)

Jingjing Wang, Ping Li, Yun Wang, Ziyi Liu, Dongqi Wang, Jianjun Liang, Qiaohui Fan**

This PDF file includes:

Experimental section

Figures S1 to S19

Table S1 and S2

References

Experimental section:

Chemicals and materials. Melamine (99%), potassium thiocyanate (KSCN, 99%), Potassium chloride, (KCl, 99.5%), Sodium chloride (NaCl, 99.9%), Ammonium chloride (NH_4Cl, 99.5%), Sodium hydroxide (NaOH, 97%), Methanol (≥99.5%) was purchased from Energy Chemical Co., Ltd (Anhui, China). $UO_2(NO_3)_2 \cdot 6H_2O$ was dissolved into deionized (DI) water to prepare U(VI) stock solution. All of the chemicals were used without any further purification.

Characterization. Powder X-ray diffraction (XRD) were obtained using X-ray diffractometer (D/Max-2400, Rigaku) with Cu Kα radiation. Fourier transform infrared (FT-IR) were recorded by Bruker ALPHA spectrometer at a resolution of 4 cm^{-1} in the range of 4000–400 cm^{-1} with KBr pellet. Scanning electron microscopy (SEM) was performed on a Hitachi S-4800 microscope. Transmission electron microscopy (TEM) was performed on a Fei Tecnai F30 microscope. X-ray photoelectron spectroscopy (XPS) was performed on an ESCALAB 250Xi spectrometer (Thermo-VG Scientific) equipped with Al Kα X-ray source. The binding energy of 284.6 eV attributed to the C-C peak was used to calibrate all the spectral features. The EVB was calculated using the following formula: $E_{NHE} = \Phi + E_{VBM} - 4.44$, where E_{NHE} and E_{VBM} represent the potentials of a NHE and the VB maximum (VBM), respectively, and the electron work function (Φ) of the used XPS analyzer is 4.00 eV [1]. Solid-state ^{13}C nuclear magnetic resonance (NMR) was ecorded on Advance Bruker 400M. Electron paramagnetic resonance (EPR) spectra were measured at room temperature on a Bruker ER200DSRC10/12. UV–vis diffuse reflectance spectra (DRS) were tested on a Simadzu UV2550 spectrophotometer. The band gap energies (Eg) were obtained from the tangent intersection points of $(Ahν)^2$ vs. photon energy (hν) according to the Tauc plots: $(αhν)^2$= A(hν-Eg) [2]. The specific surface area was analyzed by Brunauer−Emmett−Teller (BET) N_2 adsorption isotherms at 77 K using Micromeritics ASAP 2020 HD88 apparatus. Pore size distribution was calculated according to the Barrett–Joyner–Halenda (BJH) method. Photoluminescence (PL) spectra was collected under fluorescence spectrophotometer (FLS920, Edinburgh Instrument, UK) with an

excitation wavelength of 338 nm. The carrier dynamics were studied by time-resolved PL (TRPL) spectroscopy, and the average lifetime (τ) curves were deduced as: $\tau = \frac{A_1\tau_1^2+A_2\tau_2^2}{A_1\tau_1+A_2\tau_2}$ [3], where A1 and A2 represent the amplitudes, τ1 and τ2 are the corresponding lifetimes. The X-ray absorption spectroscopy (XAS) at U L_{III}-edge was acquired at BL14W1 station of the Shanghai Synchrotron Radiation Facility (SSRF, operated at 3.5 GeV with a maximum current of 220 mA). Data reduction and data analysis were performed with the aid of Athena.

Photoelectrochemical (PEC) measurements. Photoelectrochemical (PEC) measurements were performed on a CHI 660D electrochemical workstation (Shanghai Chenhua, China) with a typical three-electrode system in a closed glass reactor equipped with a quartz window for side illumination. Pt sheet and Ag/AgCl respectively acted as the counter electrode and reference electrode. 5 mg of catalyst were added into 1 mL mixed solvent (0.5 μL Nafion solution, 0.5 mL water and 0.5 mL ethyl alcohol). The prepared homogeneous catalyst after 3 h sonication was coated onto the pre-cleaned FTO substrates dropwise with the controlled coverage area of ≈1 cm^2. Then drying the substance at 80 °C to form the working electrode. Aqueous Na_2SO_4 (0.5 M) was used as electrolyte. In addition, for OCP measurement, aqueous solution containing Na_2SO_4 (0.2 M) and methanol (13 vol.%) (act as sacrificial donor) was used as electrolyte. Prior to OCP measurement, the system was purged with Ar for 2 h to remove dissolved oxygen.

Theoretical calculations. The density-functional theory (DFT) calculations were employed by using the projected augmented wave (PAW) in the Vienna Ab Initio Package (VASP). The generalized gradient approximation (GGA) with Perdew-Burke-Ernzerhof (PBE) functional was used for the exchange correlation energy. The kinetic energy cutoff of 500 eV was set for plane-wave expansion. The k-points mesh was set to 2×2×1 for structure optimization. The ultrasoft pseudopotential was adopted in all calculations. The vacuum slab of 18 Å was chosen to eliminate the periodic interference of unit cell. For the electronic structure iteration, the convergence tolerances was set as 1.0×10^{-5} eV/atom for energy, 0.03 eV/Å for the maximum ionic Hellmann–Feynman

force, 0.05 GPa for the maximum stress, and 0.001 Å for the maximum ionic displacement. The convergence criteria for self-consistent field (SCF) was set to 1.0 × 10^{-6} eV/atom.

Uranium concentration measurement. For the ppb level uranium samples, the samples were diluted with 1 ppb Indium (In)-0.5 M HNO_3 solution (In is used as internal standard), and measured by the inductively coupled plasma–mass spectrometry (ICP-MS, 8800, Agilent). The concentration of uranium at higher level was directly measured based on UV–vis spectrophotometry with Arsenazo III.

Figures:

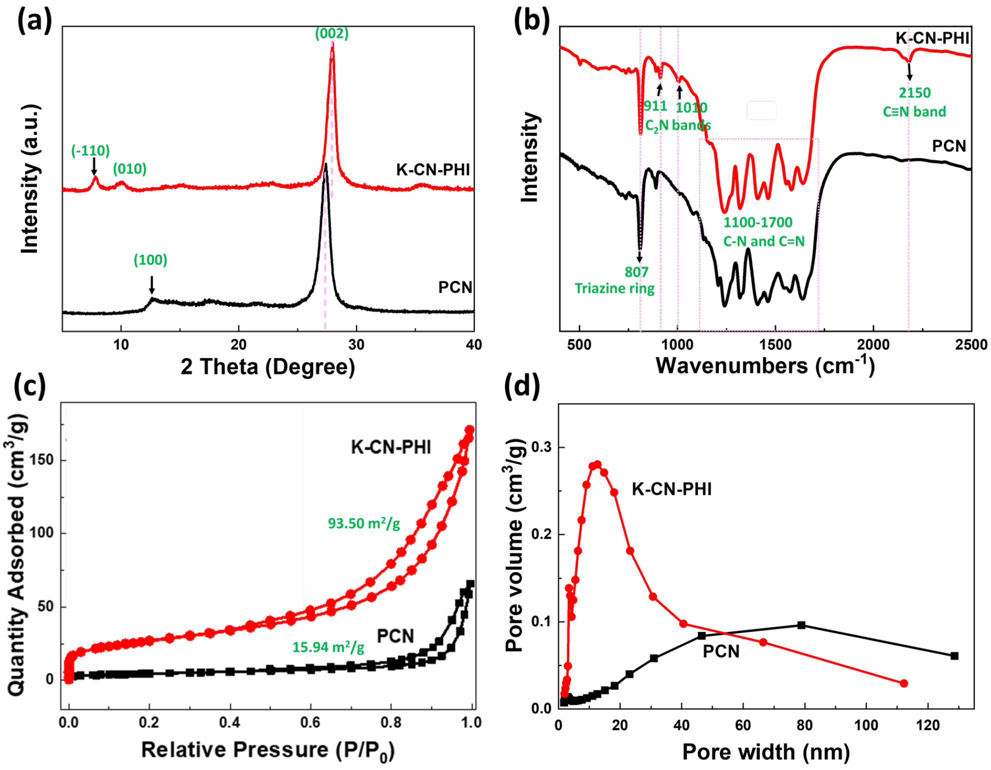

Figure S1. (a) XRD patterns, (b) FT-IR spectra, (c) BET N_2 adsorption-desorption isotherms; (d) BJH pore size distribution of PCN and K-CN-PHI.

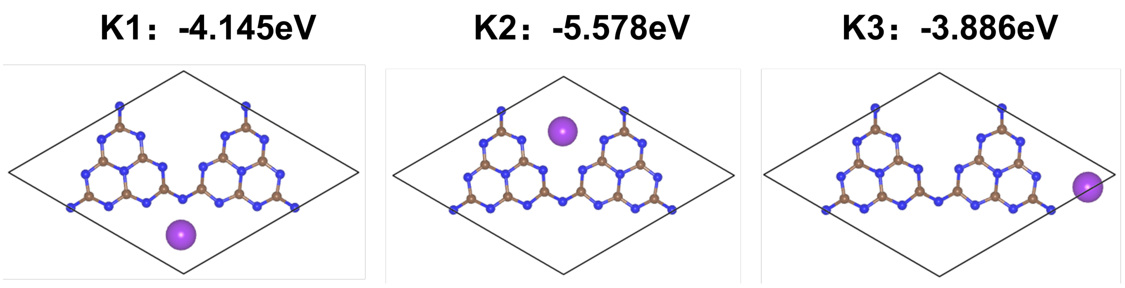

The most stable structure：

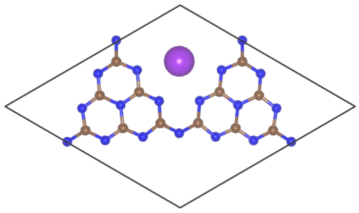

Figure S2. The initial structures and the optimized configuration K-CN-PHI.

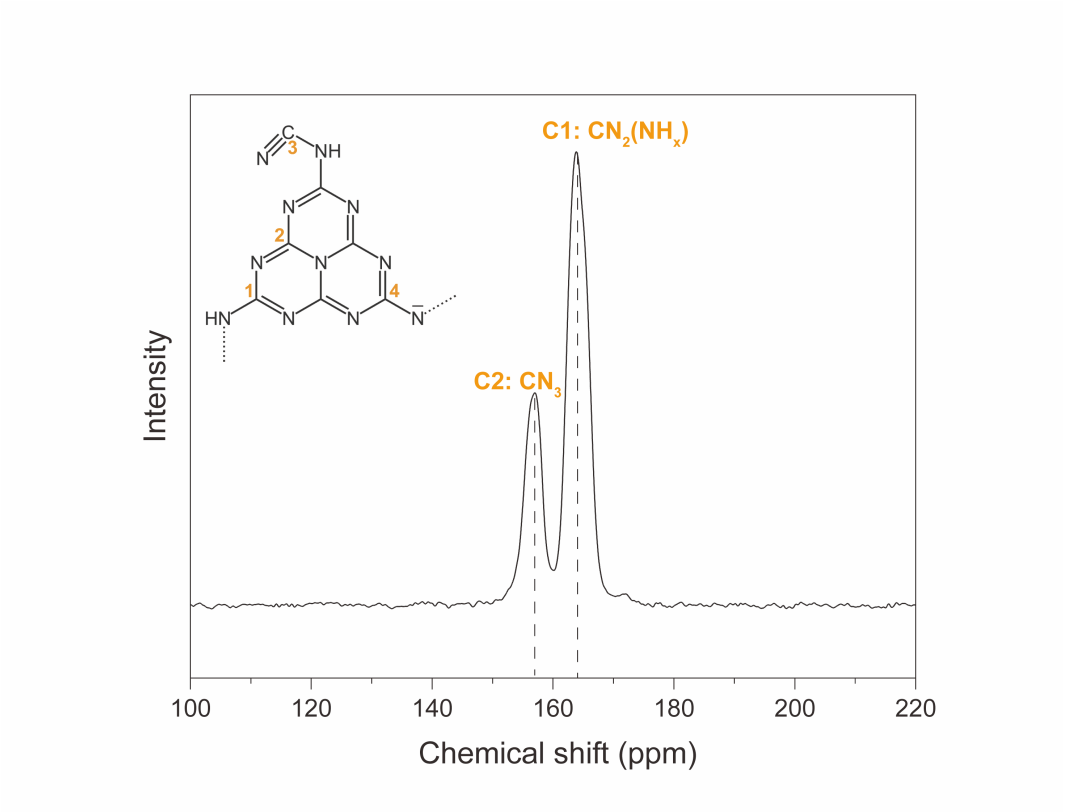

Figure S3. The solid-state ^{13}C NMR spectrum of K-CN-PHI.

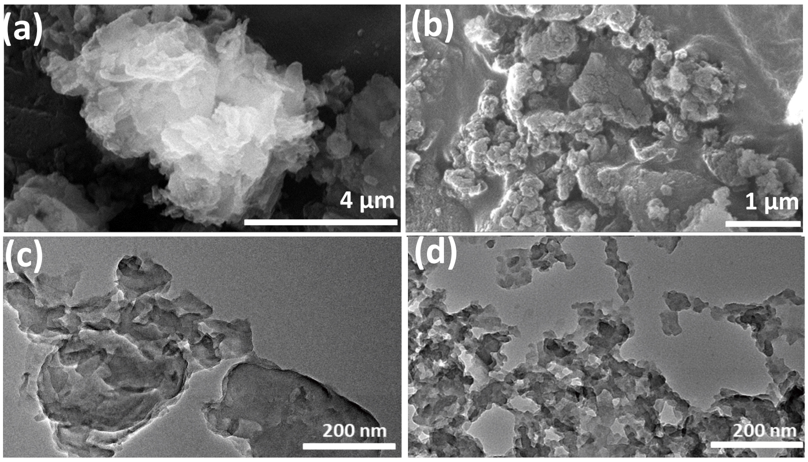

Figure S4. (a, b) SEM and (c, d) TEM images of (a, c) PCN and (b, d) K-CN-PHI.

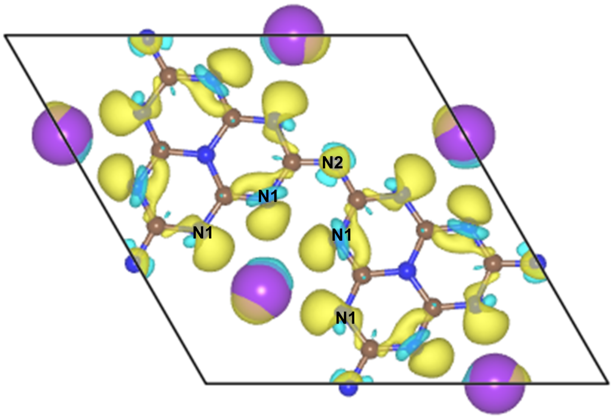

Figure S5. The charge density difference of K-CN-PHI (the yellow and cyan regions represent charge accumulation and depletion in the space; the isosurface value is 0.002 e/$Bohr^3$).

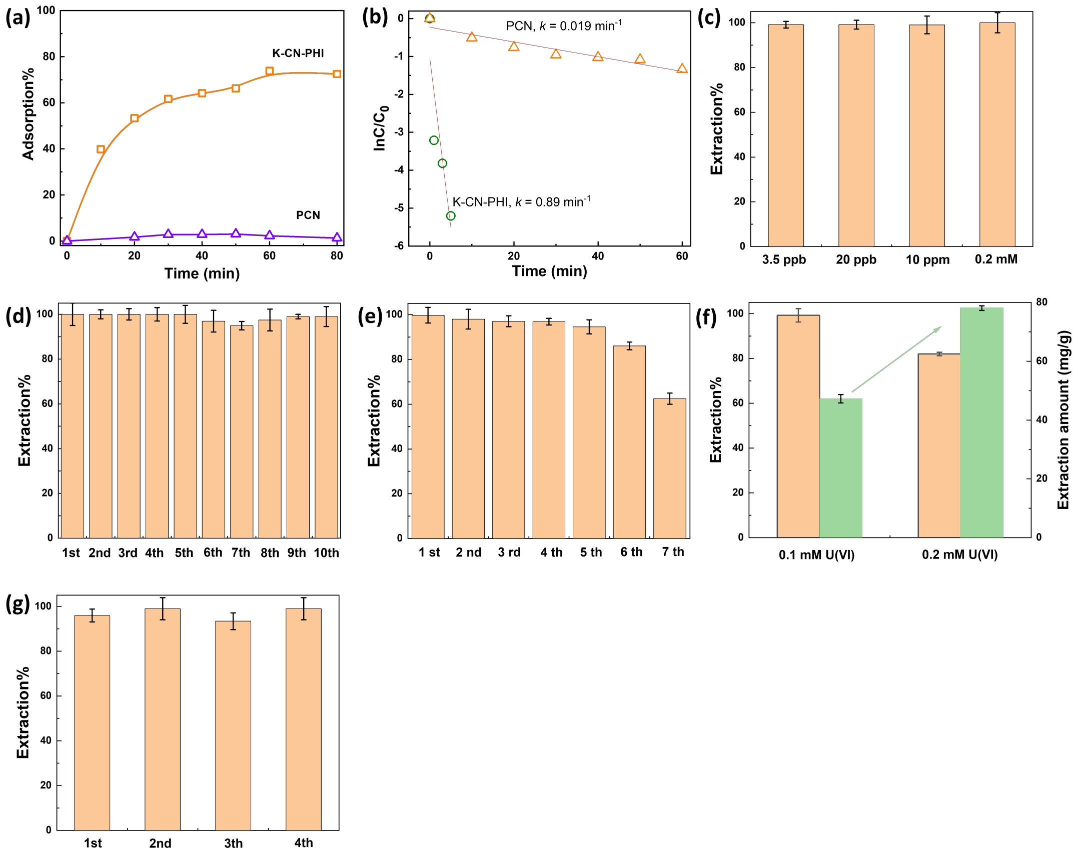

Figure S6. (a) Adsorption kinetics of U(VI) on PCN and K-CN-PHI in the dark; (b) the fitting curves of the photocatalytic kinetics of PCN and K-CN-PHI based on pseudo-first-order kinetic models (-ln(Ct/C_0) = kt); (c) the photocatalytic ability of K-C-PHI under different U(VI) concentration, where the irradiation time is 10 min, and the uranium concentration is from 3.5 ppb to 10 ppm to cover the range of uranium concentrations expected in seawater (typically 3.3 ppb) [4], groundwater (20 ppb, lower than the drinking water permissible limit of 30 μg/L by WHO) [5], and industry wastewater (10 ppm, referred to previously reported data [6]); (d) the photocatalytic ability of K-C-PHI at 1.0 mM of U(VI). Considering that uranium with high concentration may be hydrolyzed under near-neutral conditions, the extraction ability was tested by stepwise supplement of fresh U(VI) for 10 times (the uranium concentration fed each time was calculated exactly to be 0.1 mM); (e) the photocatalytic ability of K-C-PHI at 1.4 mM of U(VI), where fresh 0.2 mM of U(VI) solution was filled into reactor for 7 runs; (f) the dark reduction ability of K-CN-PHI with 0.1 mM and 0.2 mM uranium fed; (g) the dark reduction ability of K-CN-PHI for 4 runs (The

system was re-irradiated to produce the long-lived radicals, and the system would rapidly turn blue again. Then the system was tested for the next run (0.1 mM U(VI) fed for each run)).

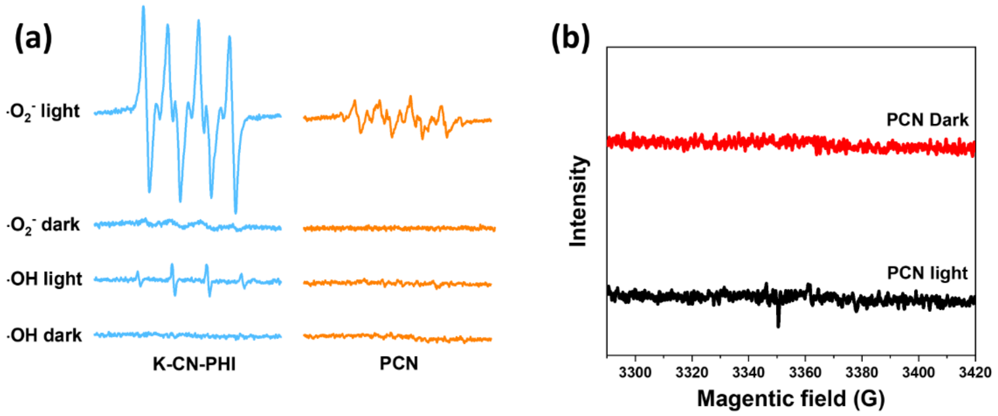

Figure S7. (a) DMPO spin-trapping EPR spectra of PCN and K-CN-PHI in the dark and under visible light irradiation; (b) EPR spectra of PCN suspension in the dark and upon irradiation.

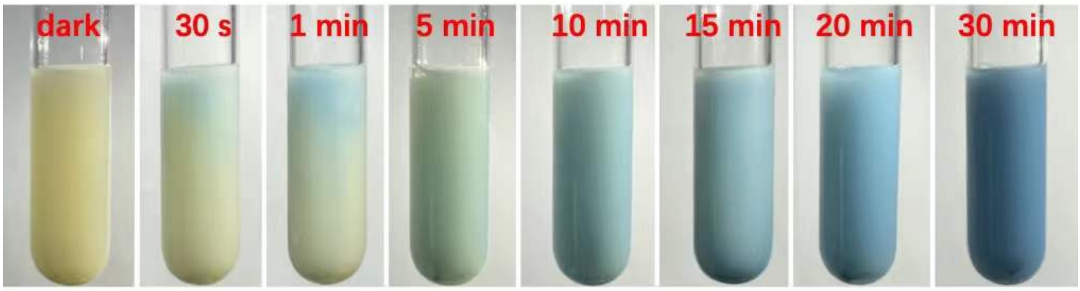

Figure S8. The color evolution of K-CN-PHI suspension under different irradiation time.

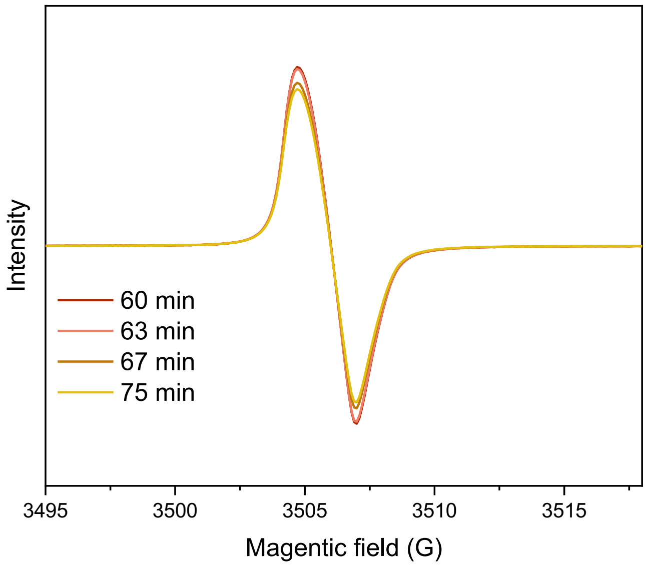

Figure S9. EPR signals of the K-CN-PHI suspension when further prolonging the irradiation time from 60 min to 75 min.

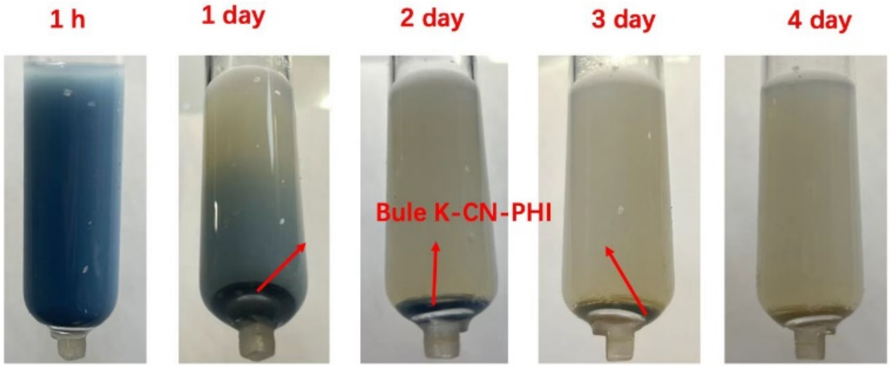

Figure S10. The color evolution of K-CN-PHI suspension under different dark time (Above is a small population of colloidal-like K-CN-PHI that is not easy to be precipitated, and the below is the K-CN-PHI precipitation).

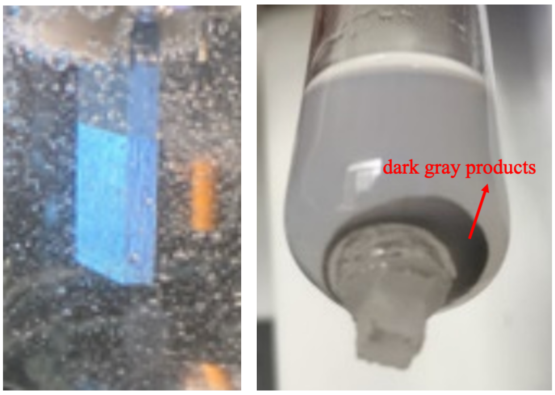

Figure S11. (a) The K-CN-PHI photoelectrodes with blue color; (b) the formed dark

gray products after dark photocatalysis.

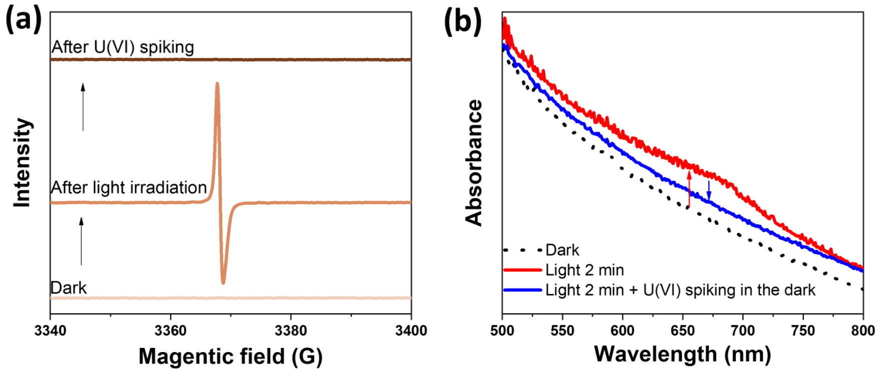

Figure S12. (a) EPR spectra of K-CN-PHI suspension; (b) UV/visible absorption spectral changes of K-CN-PHI before and after reacted with U(VI).

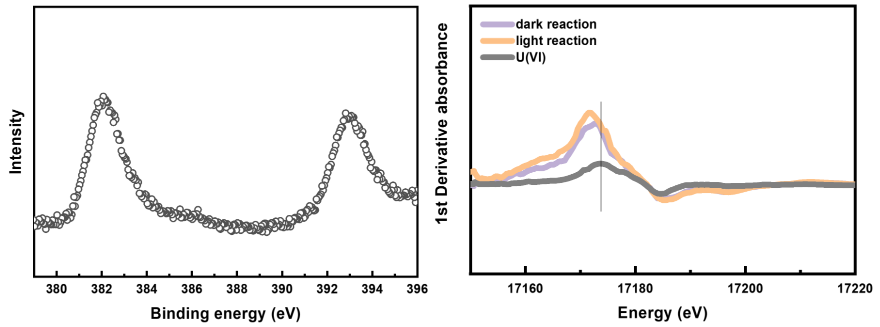

Figure S13. (a) XPS of U(VI) adsorbed-K-CN-PHI; (b) the first derivative XANES spectra of adsorbed U(VI) and the reduced products after light or dark reaction.

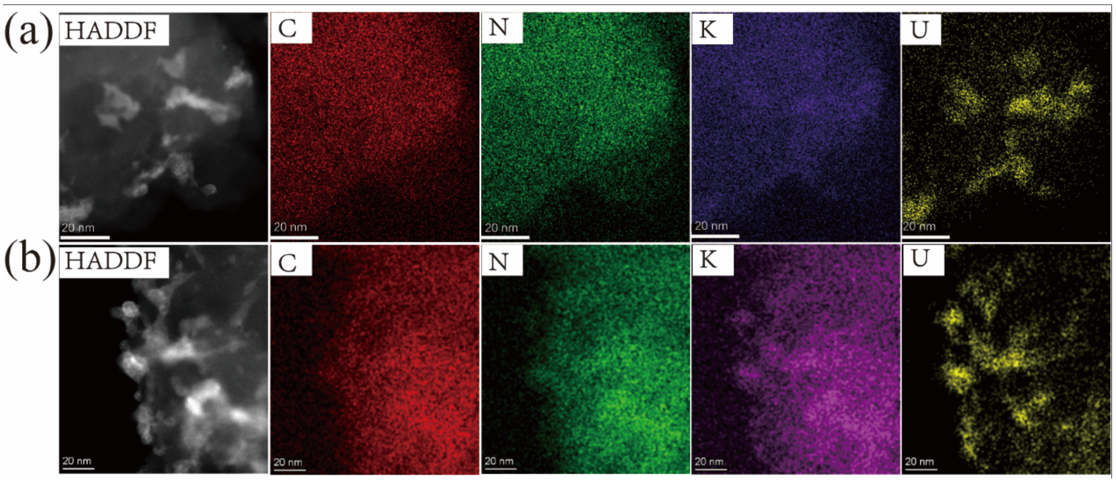

Figure S14. (a) STEM observation and Elemental distribution maps of C, N, K and U after reaction under light and (b) in darkness.

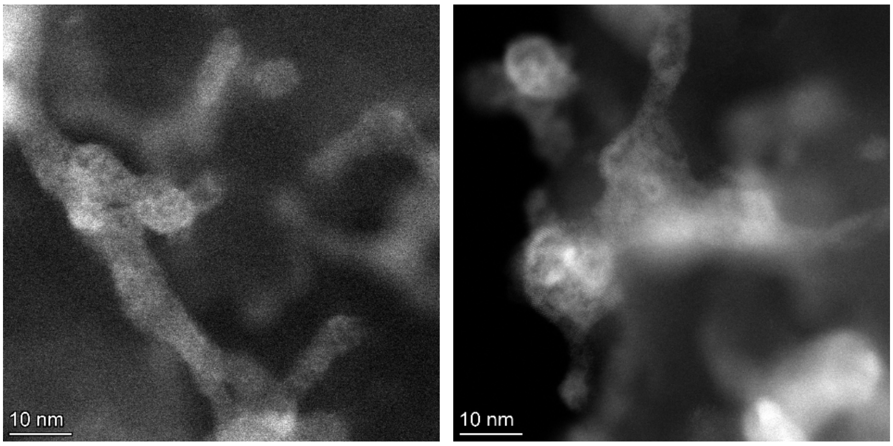

Figure S15. The HAADF-STEM images (scale bar 10 nm) of chain-like uranium oxides clusters.

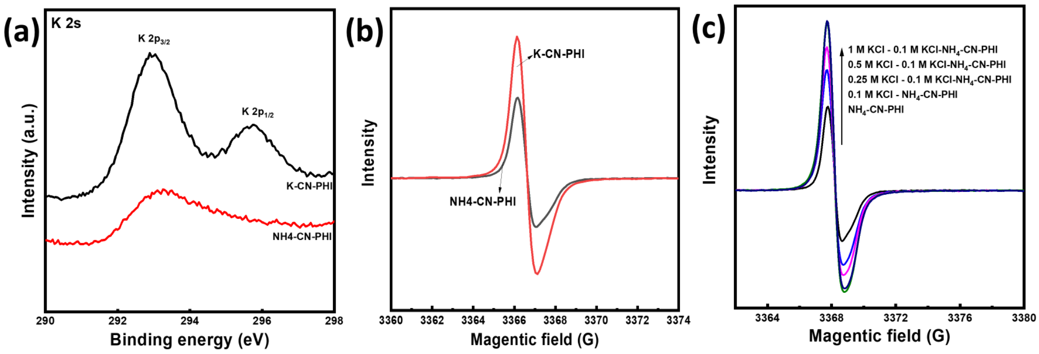

Figure S16. (a) K 2s XPS spectra for K-CN-PHI and NH_4-CN-PHI; (b) EPR spectra for K-CN-PHI and NH_4-CN-PHI; (c) EPR spectra for K-CN-PHI and NH_4-CN-PHI.

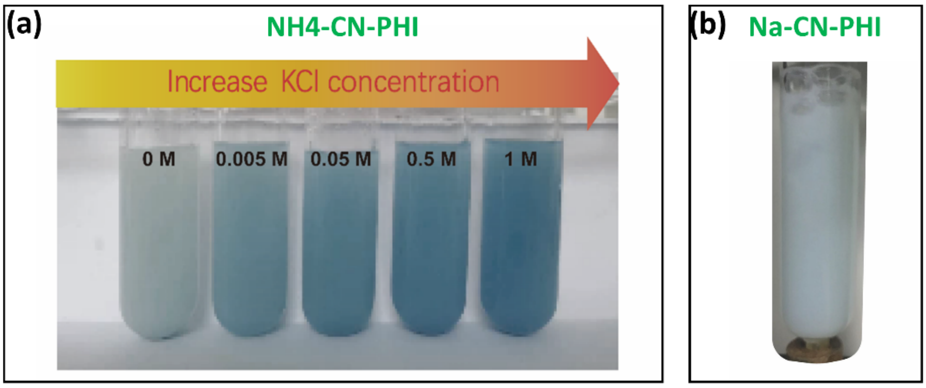

Figure S17. (a) The evolution of sample color after adding KCl into NH_4-CN-PHI suspension; (b) image of the Na-CN-PHI suspension after light irradiation.

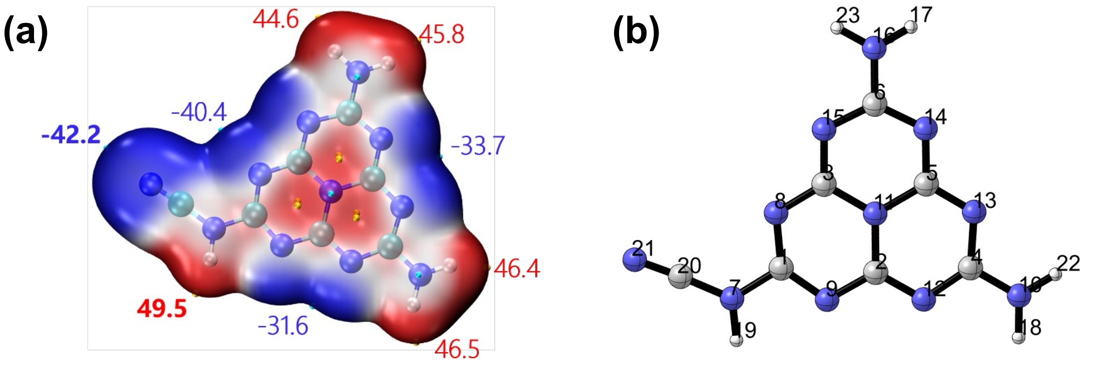

Figure S18. (a) The electrostatic potential surface of neutral CN-PHI, and the maximum and minimum points are represented by little balls colored by orange and azure blue, respectively. The ESP values of these points are also marked. (unit: kcal/mol, isovalue: 0.001). The cyano group undertakes the global maximum (49.5 kcal/mol) and minimum points (-42.2 kcal/mol) of molecules; (b) The structure and atomic indexes for the conceptual density functional theory analysis.

The role of the cyano group is confirmed by electrostatic potential surface (Figure S18a) and the result shows the cyano group undertakes the global maximum (49.5 kcal/mol) and minimum points (-42.2 kcal/mol) of molecules. The high charge polarization in small localized area can feed more electrostatically driven reactions. The role of the cyano group is also verified by conceptual density functional theory (Figure S18b, Table S2), the results show excess electron will accumulate in the nitrogen of cyano which carry the most negative charge (-0.3059). This makes the nitrogen in cyano a better nucleophilic and radical reaction site from Fukui functions (f_+, f_0). We also noticed the C attached to cyano group exhibited high electrophilicity, which is caused by the polarization of cyano group.

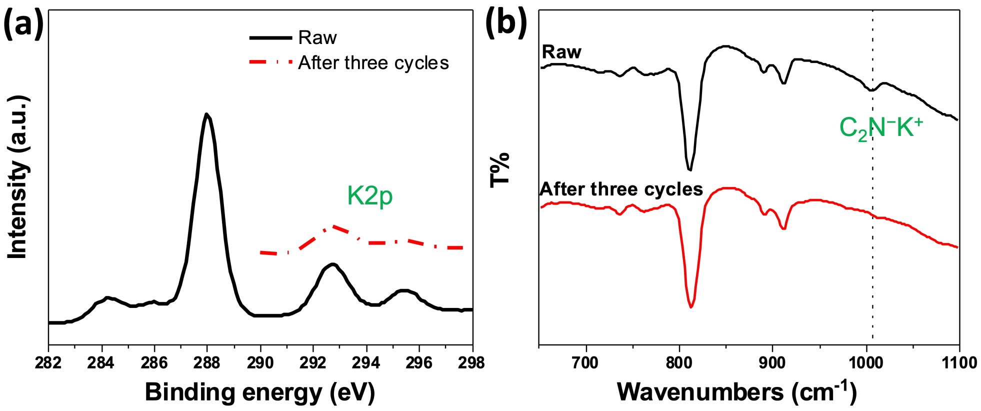

Figure S19. (a) XPS and (b) FT-IR spectra before and after mutiple light-dark cycles

(three cycles means K-CN-PHI undergo three charge-discharge processes).

Table S1 List of reported U(VI) photoreduction rate over different catalysts (modified according to reported results [35])

Catalyst	Conditions	Light source	Removal amount	Photocatalytic time(min)	Reference
TiO_2	0.21 mM U(VI), m/V= 1g/L, pH = 2.7, 1.68 mM EDTA	15 W black-light fluorescent bulb (wavelength 320~400 nm)	53%	360	[7]
TiO_2	0.168 mM U(VI), pH = 2.0, Methanol	UV light (400 W mercury discharge lamp)	98%	360	[8]
TiO_2 (P25)	0.021 mM U(VI), m/V = 0.2 g/L, 5.0 vol% Methanol	UV light (400 W mercury discharge lamp)	~98%	40	[9]
TiO_2	0.42 mM U(VI), m/V = 0.4 g/L, pH = 5.5, 200 mg/L HCOONa	UV light (400 W mercury discharge lamp)	96%	135	[10]
TiO_2	0.042 mM U(VI), m/V = 0.2 g/L, pH = 5.5, 5.0 vol% Methanol	3 W UV LED lamp (central wavelength at 365 nm)	96%	50	[11]
TiO_2	0.25 mM U(VI), m/V = 1.0 g/L, pH = 3.0, 1.0 M 2-PrOH	UV light (125 W medium pressure mercury lamp)	100%	60	[12]
TiO_2	0.1 mM U(VI), m/V = 0.6 g/L, pH = 5.0	UV light (350 W mercury discharge lamp)	100%	80	[13]
TiO_2/RGO/Fe_3O_4	0.1 mM U(VI), m/V = 0.56 g/L, pH = 4.0	UV light (100 W high-pressure mercury lamp)	~100%	30	[14]
Black TiO_2	0.2 mM U(VI), m/V = 0.25 g/L, pH = 6.0, 14 vol% Methanol	Visible light (350 W Xe lamp with a 420 nm cutoff filter)	88%	10	[15]
ZnO/rectorite	0.021 mM U(VI), m/V = 1.5 g/L, 1.0 M Methanol	UV-visible light (300 W Xe arc lamp)	98%	180	[16]
Sn-In_2S_3	0.06 mM, m/V = 0.15 g/L, pH = 6.0	Visible light (wavelength > 400 nm,	95%	40	[17]

		500 W Xe lamp)			
B-g-C_3N_4	0.12 mM U(VI), m/V = 0.5 g/L, pH = 7.0, Methanol	Visible light (500 W Xe lamp with a 420 nm cutoff filter)	93%	20	[18]
S-g-C_3N_4	0.12 mM U(VI), m/V = 0.5 g/L, pH = 7.0, 2.5 vol% Methanol	Visible light (350 W Xe lamp with a 420 nm cutoff filter)	95%	20	[19]
P-g-C_3N_4	0.12 mM U(VI), m/V = 0.5 g/L, pH = 7.0, 2.5 vol% Methanol	Visible light (350 W Xe lamp with a 420 nm cutoff filter)	84%	20	[20]
g-C_3N_4	0.084 mM U(VI), m/V = 1.0 g/L, pH = 4.0, 10 mg/L Cr(VI), 20 ppm bisphenol A	Visible light (350 W Xe lamp with a 420 nm cutoff filter)	97%	30	[21]
π-e^--incorporated g-C_3N_4	0.168 mM U(VI), m/V = 1.0 g/L, pH = 5.0, in air	Visible light (350 W Xe lamp with a 420 nm cutoff filter)	95%	80	[22]
Isotype g-C_3N_4	0.168 mM U(VI), m/V = 1.0 g/L, pH = 5.28, 4.0 vol% Methanol	Visible light (300 W Xe lamp, wavelength ≥ 420 nm)	98%	30	[23]
PCB/CN	0.42 mM U(VI), m/V = 0.5 g/L, pH = 4.0	Visible light (300 W Xe lamp, wavelength ≥ 400 nm)	100%	120	[24]
g-C_3N_4/TiO_2	0.084 mM U(VI), m/V = 0.25 g/L, pH = 6.9, 20 mg/L As(III)	UV-visible light (wavelength 320-780 nm, 300 W Xe lamp)	99%	25	[25]
mpg-g-C_3N_4	0.1 mM U(VI), m/V = 0.5 g/L, pH = 6.0, 14 vol% Methanol	Visible light (350 W Xe lamp with a 420 nm cutoff filter)	100%	20	[26]
Ti_3C_2/$SrTiO_3$	0.21 mM U(VI), m/V = 0.33 g/L, pH = 4.0	UV-visible light (wavelength 320-2500 nm, 300 W Xe lamp)	77%	180	[27]
GO/KTO	0.21 mM U(VI), m/V = 1.0 g/L, pH = 6.0	UV-visible light (500 W Xe lamp)	98%	120	[28]
Niobate/Titanate	0.084 mM U(VI), m/V = 0.2 g/L, pH = 5.0	Simulated solar light (450 W xenon lamp)	89%	240	[29]

$CaTiO_3$	0.084 mM U(VI), m/V = 0.2 g/L, pH = 7.5	Visible light (300 W Xe lamp, wavelength ≥ 380nm)	98.2%	180	[30]
$ZnFe_2O_4$	0.21 mM U(VI), m/V = 0.2 g/L, pH = 5.0, 24.0 mM CH_3OH	Visible light (300 W Xe lamp with a 420 nm cutoff filter)	>95%	40	[31]
MIL-53 (Fe)	0.21 mM U(VI), m/V = 0.4 g/L, pH = 4.5, 1.0 mM HCOOH	Visible light (285 W Xe lamp, wavelength ≥ 420 nm)	80%	120	[32]
$SnO_2/CdCO_3/CdS$	0.21 mM U(VI), m/V = 0.4 g/L, pH = 4.0	Visible light (500 W Xe lamp with a 420-nm cut-off filter)	~ 75%	70	[33]
Carboxylated g-C_3N_4	0.1 mM U(VI), m/V = 0.4 g/L, pH = 8.2， 2 mM HCO_3^{2-}, 14 vol% Methanol	Visible light (350 W Xe lamp with a 420-nm cut-off filter)	100%	10	[34]
CdS/g-C_3N_4	0.1 mM U(VI), m/V = 1.0 g/L, pH = 6.0	Visible light (350 W Xe lamp with a 420 nm cutoff filter)	100%	6	[35]
CdS/TiO_2	0.21 mM U(VI), m/V = 0.4 g/L, pH = 6.0, 5 vol% Methanol	solar simulator with a 420 nm cut-off filter	97%	240	[36]
Ag-doped Sn S2@$InVO_4$	0.25 mM U(VI), m/V = 0.25 g/L, pH = 6.0	Visible light (450 W Xe lamp with a 400 nm cutoff filter)	98%	100	[37]
ZIF-8/g-C_3N_4	0.042 mM U(VI), m/V = 0.1 g/L, pH = 6.0, Ethanol	Visible light (300 W Xe lamp with a 420 nm cutoff filter)	100%	30	[38]
PN-PCN-222	1.68 mM U(VI), m/V = 0.5 g/L, pH = 6.0, 10 vol% Methanol	Visible light (350 W Xe lamp with a 420 nm cutoff filter)	97%	1440	[39]
Ag/ZIF-8	0.84 mM U(VI), m/V = 0.25 g/L, pH = 5.0	Xe lamp	93%	60	[40]
MOF/Black Phosphorus Quantum Dots	0.067 mM U(VI), m/V = 5 mg/L, pH = 8.0	Simulated sunlight (300W Xe lamp)	15%	720	[41]

PT-BN-AO	0.042 mM U(VI), m/V = 5 mg/L, pH = 5.0	300W Xe lamp (320 -780 nm)	46%	2100	[42]
NDA-TN-AO	0.042 mM U(VI), m/V = 5 mg/L, pH = 5.0	300W Xe lamp (320 -780 nm)	29%	2100	[43]
TP-TMT	2.1 mM U(VI), m/V = 0.125 g/L, pH = 5.0, 20 mM methanol	300 W xenon lamp with a 420 nm cutoff filter	59%	300	[44]
DHBD-TMT	2.1 mM U(VI), m/V = 0.125 g/L, pH = 5.0, 20 mM methanol	A300 W Xe lamp (320 nm-780 nm)	66%	300	[45]
SCN-19	0.2 mM U(VI), m/V = 0.4 g/L, pH = 6.0, 5 vol% Methanol	Visible light (300 W Xe lamp with a 420 nm cutoff filter)	91%	3000	[46]
	0.2 mM U(VI), m/V = 0.4 g/L, pH = 4.0	Visible light (350 W Xe lamp with a 420 nm cutoff filter)	95%	80	This work
K-CN-PHI	0.2 mM U(VI), m/V = 0.4 g/L, pH = 6.0, 3 vol% Methanol	Visible light (350 W Xe lamp with a 420 nm cutoff filter)	99%	5	This work
	0.1 mM U(VI), m/V = 0.5 g/L, pH = 6.0	Dark photocatalysis	98%	1	This work

Table S2. The atomic Hirshfeld charges for neutral (q(N)), rich electron radical (q(N+1)), poor electron radical (q(N-1)), Fukui functions f_+ (nucleophilic reaction), and f_0 (radical reaction) index for structure in Figure S18b.

Atom	q(N)	q(N+1)	q(N-1)	f_+	f_0
**1(C)**	**0.1659**	**0.0774**	**0.1930**	**0.0885**	**0.0578**
2(C)	0.1614	0.1077	0.1836	0.0537	0.0380
3(C)	0.1650	0.1134	0.1858	0.0516	0.0362
4(C)	0.1623	0.1057	0.1900	0.0566	0.0422
5(C)	0.1596	0.1308	0.1821	0.0288	0.0256
6(C)	0.1635	0.1083	0.1907	0.0552	0.0412
**7(N)**	**-0.0463**	**-0.0787**	**-0.0220**	**0.0324**	**0.0283**
8(N)	-0.2034	-0.2436	-0.1186	0.0401	0.0625
9(N)	-0.2180	-0.2601	-0.1182	0.0421	0.0710
10(N)	-0.1247	-0.1781	-0.0831	0.0534	0.0475
11(N)	-0.0191	-0.0613	-0.0180	0.0422	0.0217
12(N)	-0.2198	-0.2586	-0.1250	0.0388	0.0668
13(N)	-0.2214	-0.2636	-0.1250	0.0422	0.0693
14(N)	-0.2207	-0.2627	-0.1284	0.0419	0.0671
15(N)	-0.2141	-0.2521	-0.1268	0.0380	0.0627
16(N)	-0.1229	-0.1759	-0.0812	0.0529	0.0473
17(H)	0.1464	0.1187	0.1697	0.0277	0.0255
18(H)	0.1459	0.1190	0.1695	0.0269	0.0252
19(H)	0.1619	0.1395	0.1791	0.0224	0.0198
**20(C)**	**0.1057**	**0.0808**	**0.1161**	**0.0250**	**0.0177**
**21(N)**	**-0.2206**	**-0.3059**	**-0.1539**	**0.0853**	**0.0760**
22(H)	0.1461	0.1182	0.1698	0.0279	0.0258
23(H)	0.1473	0.1210	0.1707	0.0263	0.0248

References

1. H. Yu, R. Shi, Y. Zhao, T. Bian, Y. Zhao, C. Zhou, G. Waterhouse, L. Wu, C. Tung, T. Zhang, *Adv. Mater.* **2017**, 29, 1–7.
2. P. Makuła, M. Pacia, W. Macyk, *J. Phys. Chem. Lett*. **2018**, 9, 6814–6817.
3. B. Gupta,V. Rathee, T. Narayanan, P. Thanikaivelan, A. Saha, Govind, S. Singh, V. Shanker, A. Marti, P. Ajayan, *Small* **2011**, 7, 1767-1773.
4. C. Abney, O. R. Mayes, T. Saito, S. Dai, Materials for the recovery of uranium from seawater, *Chem. Rev*. **2017**, 117, 13935–14013.
5. WHO, Guidelines for Drinking Water Quality, Fourth Edition, *World Health Organization*, Geneva **2011**.
6. Y. Ye, J. Jin, Y. Liang, Z. Qin, X. Tang, Ya. Feng, Miao. Lv, S. Miao, C. Li, Y. Chen, F. Chen, Y. Wang, Efficient and durable uranium extraction from uranium mine tailings seepage water via a photoelectrochemical method. *Iscience,* **2021**, 24, 103230.
7. J. Chen, D. Ollis, W. Rulkens, H. Bruning, *Colloid. Surface. A* **1999**, 151, 339–349.
8. C. Evans, G. Nicholson, D. Faith, M. Kan, *Green Chem*. **2004**, 6, 196–197.
9. L. Zhang, H. Li, L. Li, J. Deng, W. Deng, Y. Zhao, *Chem. Lett.* **2013**, 42, 689–690.
10. G. Wang, J. Zhen, L. Zhou, F. Wu, N. Deng, *J. Radioanal. Nucl. Chem.* **2015**, 304, 579–585.
11. L. Li, H. Li, W. Deng, B. Qin, Z. Fan, Y. Zhao, *Chem. Lett.* **2014**, 43, 936–937.
12. V. Salomone, J. Meichtry, G. Zampieri, M. Litter, *Chem. Eng. J.* **2015**, 261, 27–35.
13. P. Li, J. Wang, Y. Wang, J. Liang, B. He, D. Pan, Q. Fan, X. Wang, *Chem. Eng. J.* **2019**, 365, 231–241.

14. Z. Li, Z. Huang, W. Guo, L. Wang, L. Zheng, Z. Chai, W. Shi, *Environ. Sci. Technol.* **2017**, 51, 5666–5674.
15. J. Wang, Y. Wang, W. Wang, T. Peng, J. Liang, P. Li, D. Pan, Q. Fan, W. Wu, *Environ. Pollut.* **2020**, 262, 114373.
16. Y. Guo, L. Li, Y. Li, Z. Li, X. Wang, G. Wang, *J. Radioanal. Nucl. Chem.* **2016**, 310, 883-890.
17. J. Feng, Z. Yang, S. He, X. Niu, T. Zhang, A. Ding, H. Liang, X. Feng, *Chemosphere* **2018**, 212, 114–123.
18. C. Lu, R. Chen, X. Wu, M. Fan, Y. Liu, Z. Le, S. Jiang, S. Song, *Appl. Surf. Sci.* **2016**, 360, 1016–1022.
19. C. Lu, P. Zhang, S. Jiang, X. Wu, S. Song, M. Zhu, Z. Lou, Z. Li, F. Liu, Y. Liu, Y. Wang, Z. Le, *Appl. Catal. B: Environ.* **2017**, 200, 378–385.
20. X. Wu, S. Jiang, S. Song, C. Sun, *Appl. Surf. Sci*. **2018**, 430, 371–379.
21. H. Wang, H. Guo, N. Zhang, Z. Chen, B. Hu, X. Wang, *Environ. Sci. Technol.* **2019**, 53, 6454–6461.
22. J. Gong, Z. Xie, C. Xiong, C. Liu, Z. Li, Z. Le, *J. Radioanal. Nucl. Chem.* **2019**, 322, 1115–1125.
23. Z. Le, C. Xiong, J. Gong, X. Wu, T. Pan, Z. Chen, Z. Xie, *Environ. Pollut.* **2020**, 260, 114070.
24. F. Yu, Z. Yu, Z. Xu, J. Xiong, Q. Fan, X. Feng, Y. Tao, J. Hua, F. Luo, *Mol. Syst. Des. Eng.* **2020**, 5, 882–889.
25. X. Jiang, Q. Xing, X. Luo, F. Li, J. Zou, S. Liu, X. Li, X. Wang, *Appl. Catal. B: Environ*. **2018**, 228, 29–38.

26. J. Wang, Y. Wang, W. Wang, Z. Ding, R. Geng, P. Li, D. Pan, J. Liang, H. Qin, Q. Fan, *Chem. Eng. J.* **2020**, 383, 123193.
27. H. Deng, Z. Li, L. Wang, L.Y. Yuan, J. Lan, Z. Chang, Z. Chai, W. Shi, *ACS Appl. Nano Mater.* **2019**, 2, 2283–2294.
28. M. Zhu, Y. Cai, S. Liu, M. Fang, X. Tan, X. Liu, M. Kong, W. Xu, H. Mei, T. Hayat, *Environ. Pollut*. **2019**, 248, 448–455.
29. X. Liu, P. Du, W. Pan, C. Dang, T. Qian, H. Liu, W. Liu, D. Zhao, *Appl. Catal. B: Environ.* **2018**, 231, 11–22.
30. S. Lu, K. Zhu, T. Hayat, N. Alharbi, C. Chen, G. Song, D. Chen, Y. Sun, *J. Hazard. Mater.* **2019**, 364, 100-107.
31. P. Liang, L. Yuan, H. Deng, X. Wang, L. Wang, Z. Li, S. Luo, W. Shi, *Appl. Catal. B: Environ.* **2020**, 267, 118688.
32. Z. Yan, H. Xi, L. Yuan, *Environ. Sci.* **2019**, 40, 1819–1825 (In Chinese).
33. Y. Zhang, M. Zhu, S. Zhang, Y. Cai, Z. Lv, M. Fang, X. Tan, X. Wang, *Appl. Catal. B Environ.* **2020**, 279, 119390.
34. P. Li, Y. Wang, J. Wang, L. Dong, W. Zhang, Z. Lu, J. Liang, D. Pan, Q. Fan, *Chem. Eng. J.* **2021**, 414, 128810.
35. P. Li, J. Wang, Y. Wang, L. Dong, W. Wang, R. Geng, Z. Ding, D. Luo, D. Pan, J. Liang, Q. Fan, *Chem. Eng. J.* **2021**, 425, 131552.
36. C. Yu, Z. Zhang, Z. Dong, Y. Xiong, Y. Wang, Y. Liu, X. Cao, W. Dong, M. Liu, Y. Liu, *J. Solid State Chem.* **2021**, 298, 122053.
37. S. He, Z. Yang, X. Cui, X. Zhang, X. Niu, *Chemosphere*, **2020**, 260, 127548.
38. M. Qiu, Z. Liu, S. Wang, B. Hu, *Environ. Res.* **2021**, 196, 110349.

39. L. Hui, F. Zhai, D. Gui, X. Wang, C. Wu, Z. Duo, D. Xing, D. Hong, X. Su, D. Juan, L. Zhang, Z. Chai, W. Shuao, *Appl. Catal. B Environ.* 2019, 254, 47-54.
40. P. Jiang, K. Yu, H. Yuan, R. He, M. Sun, F. Tao, L. Wang, W. Zhu, *J. Mater. Chem. A* **2021**, 9, 9809-9814.
41. M. Chen, T. Liu, X. Zhang, R. Zhang, S. Tang, Y. Yuan, Z. Xie, Y. Liu, H. Wang, K. Fedorovich, N. Wang, *Adv. Funct. Mater*. **2021**, 31, 2100106.
42. W. Cui, F. Li, R. Xu, C. Zhang, X. Chen, R. Yan, R. Liang, J. Qiu, *Angew. Chem. Int. Edit.* **2020**, 59, 17684-17690.
43. W. Cui, C. Zhang, R. Xu, X. Chen, J. Qiu, *ACS ES&T Water* **2020**, 1, 440-448.
44. R. Xu, W. Cui, C. Zhang, X. Chen, W. Jiang, R. Liang, J. Qiu, *Chem. Eng. J.* **2021**, 419, 129550.
45. W. Cui, C. Zhang, R. Xu, X. Chen, W. Jiang, Y. Li, R. Liang, L. Zhang, J. Qiu, *Appl. Catal. B Environ*. **2021**, 194, 120250.
46. H. Zhang, W. Liu, A. Li, D. Zhang, X. Li, F. Zhai, L. Chen, L. Chen, Y. Wang, S. Wang, *Angew. Chem. Int. Ed*. **2019**, 58, 16110–16114.
